# Supplementary material for: Acceptance and compliance with micronutrient powder (MNP) among children aged 6–23 months in northern Nigeria
Source: PLOS Glob Public Health. 2022 Oct 17;2(10):e0000961. doi: 10.1371/journal.pgph.0000961 (PMC10022258; doi:10.1371/journal.pgph.0000961)
Supplement: S1 Dataset — (ZIP) [file pgph.0000961.s008.zip › S8_Dataset/Direct Observations.zip!/Direct Observations/280615_K_DW_SU_1_DO.pdf]

### 1.1.1 Instrument F –Direct Observation Form

| |
|---|
| **<<Instrument F Direct Observation Form>>** |
| **PI Name:** PragyaMathema,Stephen Kodish<br>**Study Title:** Formative research to develop an infant and young child feeding (IYCF) intervention with micronutrient powder (MNP) for children aged 6 – 23 months<br>**PI Version/Date:** Version1.0/January 24, 2015 |

Date: 28/06/15 Location: Didogo

Data Collector: Samira Usman

| |
|---|
| Main goals of this direct observation are to answer the following questions:<br><br>1. In this household, what would be the best way(s) to give nutritional supplements to young children? Why?<br><br>2. In this household, describe the food sharing that you observe throughout the meal. Are there any predictable patterns?<br><br>3. In this household, who is favored and how is this favoritism expressed during meal preparation and eating? |

Household Composition Table (Fill out as you do data collection)

| INITIALS | SEX (M/F) | AGE (estimated) | Relationship to household | Description |
|---|---|---|---|---|
| MM | F | 32yrs | Mother | Average height dark wearing Ankara wrapper shirt |
| BB | F | 20months | Daughter | Small girl wearing blue top and short skirts |
| SS | F | 10yrs | Daughter sister to the baby | Fair girl wearing a green wrapper and head tie with black necklace |
| TT | F | 2yrs | Neighbor daughters (twins) | They are wearing blue green |
| TS | F | 2yrs | Neighbor daughters (twins) | Ankara skirts and blouse |
| TB | M | 6yrs | Son | Young boy wearing ash short and green top |
| JS | F | 8yrs | Daughter | Dark young girl wearing green wrapper |
| FS | M | 18yrs | Son | Dark tall boy wearing black trouser and stripe green short |
| BG | F | 16yrs | Daughter | Tall dark girl wearing red Ankara |

| | | | | |
|---|---|---|---|---|
| | | | | wrapper with green short and black cap |
| FF | M | 45yrs | Father | Average height, dark wearing purple short, milk short and long black trousers on his neck |

| TIME | ACTOR INITIALS | RECIPIENT INITIALS | WHAT HAPPENED | FOOD | Reactivity |
|---|---|---|---|---|---|
| 9:00 | BB,MM | | Mother is sitting on the floor and the baby is playing with a dock baby | | |
| 9:05 | BB | | Baby is playing | | |
| 9:10 | BB | | Baby still playing | | |
| 9:15 | BB | | The baby is drinking water she fetch from the pot | | |
| 9:20 | BB | | The baby is playing | | |
| 9:25 | MM | | The mother is sweeping her room | | |
| 9:30 | BB | | Some neighbor children came into the house and call her and they went out | | |
| 9:35 | BB | | The baby is playing under the mango tree with neighbor children | | |
| 9:40 | BB | | The baby is still playing | | |
| 9:45 | MM | BB | The baby said to her mother she wants pap and the mother gave her in a cup | pap | |
| 9:50 | BB | | The baby requested for more and the mother gave her | pap | |
| 9:55 | MM | BB | The mother fetch water in a bucket and bath her | | |
| 10:00 | BB | | Baby playing with neighbor children under the mango tree | | |
| 10:05 | BB | | They are still playing | | |
| 10:10 | BB | | The baby went back and climb her mother's back | | |
| 10:15 | SS | | The sister comes into the house and the baby run to her | | |
| 10:20 | SS,BB | | The sister is singing and the baby is | | |

| TIME | ACTOR INITIALS | RECIPIENT INITIALS | WHAT HAPPENED | FOOD | Reactivity |
|---|---|---|---|---|---|
| | | | imitating her | | |
| 10:25 | SS,BB | | The sister is playing with the baby and the mother is lying down on a mat | | |
| 10:30 | BB | | The baby is playing with her doll baby | | |
| 10:35 | BB | | The baby is playing on her mother's back | | |
| 10:40 | BB | | The baby is playing with her doll baby and the mother is sleeping on a mat | | |
| 10:45 | SS | BB | The sister is helping the baby to back her doll baby | | |
| 10:50 | BB | | The baby is backing her doll baby and equally playing with sand | | |
| 10:55 | BB | | The baby is drinking water | | |
| 11:00 | BB | | The baby is sleeping on a mat, the mother is sleeping | | |
| 11:05 | MM | TT | Two small girls (twins) enter the house, the mother gave them tuwon dawa with dried baobab leaves soup, she told me that they are their neighbors, the their mother left the food with her, they eat without washing their hands | Tuwon dawa with dried baobab leaves soup | |
| 11:10 | SS, | BB | The sister gives the baby tuwon dawa with dried baobab leaves soup in a different bowl and the baby starts eating without washing her hands | Tuwon dawa with dried baobab leaves soup | |
| 11:15 | SS,BB | | The twins went out, the sister is playing with the baby | | |
| 11:20 | BB,TT,TS | | The baby is playing with the twins | | |
| 11:25 | BB,TT,TS | | They are still playing under the mango tree | | |
| 11:30 | SS | | The sister is playing with my shoes and looking at me | Playing with my | |

| TIME | ACTOR INITIALS | RECIPIENT INITIALS | WHAT HAPPENED | FOOD | Reactivity |
|---|---|---|---|---|---|
| | | | | shoes | |
| 11:35 | BB, TT, TS | | The baby and the twins are playing | | |
| 11:40 | BB,TT TS | | The baby and the twins are drinking water | | |
| 11:45 | BB,TT SS | | The baby and the twins are still playing | | |
| 11:50 | TT,BB TS | | They are still playing running around the house | | |
| 11:55 | MM,SS | | The mother and the sister are sleeping on a mat | | |
| 12:00 | | | They are still sleeping | | |
| 12:05 | BB | | The baby is sitting down trying to tie head tie for her doll baby | | |
| 12:10 | BB | | The baby is playing | | |
| | BB | | The baby is drinking water | | |
| 12:15 | BB | | The baby is playing with her doll baby | | |
| | MM,SS | | The mother and the daughter are still sleeping on a mat | | |
| 12:20 | SS | | The sister wakes up, wash her face and drank water | | |
| 12:25 | BB | | The baby is bathing her doll baby and gave it to one of the twins to hold | | |
| 12:30 | YB,BB SS | | The young boy comes back and lies on a mat.<br>The baby starts crying she wants to collect her doll baby from the twins and the they are refusing her, the sister collected and gave it to her | | |
| 12:35 | SS | BB | The sister gave the baby pap | Millet pap | |
| 12:40 | BB | | The baby is still drinking the pap, she adds water in the bowl and drank the water | | |
| 12:45 | YB | | The young boy is eating tuwon dawa with dried baobab leaves soup | Tuwon dawa | |

| TIME | ACTOR INITIALS | RECIPIENT INITIALS | WHAT HAPPENED | FOOD | Reactivity |
|---|---|---|---|---|---|
| | | | | with dried baobab soup | |
| 12:50 | JS,BB MM | | The younger sister came and climbs the mango tree.<br>The baby starts crying and the mother woke up and asks the baby to come, the mother sat her down on her lap petting her.<br>The mother enters the toilet | | |
| 12:55 | MM | BB | Mother is bathing the baby with sponge and soap including her teeth, the baby is crying.<br>Mother gives the baby some fruit in a bowl, it is a Shea butter fruits | Shea butter fruits (kade) | |
| 1:00 | FS | | The first son come back home with his hoe on his back.<br>The boy went to the room took some sorghum and gave to chickens and went out. | | |
| 1:05 | SS.BB | | The sister is eating tuwon dawa and dried baobab leaves soup in a silver bowl without washing her hands.<br>The baby is still eating the fruits | Tuwon dawa with dried baobab soup | |
| 1:10 | BB,MM | | The baby is playing with the seeds of the fruit in a bowl.<br>Mother is sitting down on a mat | | |
| 1:15 | | | Mother is losing the baby's hair while the baby is lying down on her lap | | |
| 1:20 | MM | BB | Mother still loosen the baby's hair | | |
| 1:25 | JS,YB SS | | The children are playing under the mango tree | | |
| 1:30 | BB | | The baby is sleeping on her mother's lap | | |
| 1:35 | MM | BB | Mother applying Vaseline on the baby's hair | | |

| TIME | ACTOR INITIALS | RECIPIENT INITIALS | WHAT HAPPENED | FOOD | Reactivity |
|---|---|---|---|---|---|
| 1:40 | BG | | One of the daughter comes home with a bunch of wood on her head, she drop the wood and drank water | | |
| 1:45 | MM | BB | Mother is platting the baby's hair while the baby is sleeping | | |
| 1:50 | BG | | The big girl took tuwo dawa put it in a bowl and add sugar, mixed it and drank | Tuwon dawa mixed with sugar and water | |
| 1:55 | MM | BB | Mother still platting the baby hair and the baby is sleeping | | |
| 2:00 | YB,MM | | The small boy is eating shea butter fruits (kade).<br>Mother still platting the baby's hair | Shea butter fruit | |
| 2:05 | JS,SS | | The two sisters took tuwon dawa from the roof in a bowl, take small from it, add salt and a magi, then mix it and eat without washing their hands | Tuwon dawa mixed with salt and magi | |
| 2:10 | YB | | The young boy took small from the tuwon dawa, add water and sugar mix and drank | Tuwon dawa mixed with sugar and water | |
| 2:15 | BB | | The baby is still sleeping | | |
| 2:20 | BB | | Baby sleeping | | |
| 2:25 | MM | | The mother is lying down on a local bed under the mango tree | | |
| 2:30 | BG,YB SS | | The children are sitting under the mango tree shade | | |
| 2:35 | BB | | Baby sleeping | | |
| | BG,TT,TS | | The big girl is repairing a touch light. | Tuwon | |

| TIME | ACTOR INITIALS | RECIPIENT INITIALS | WHAT HAPPENED | FOOD | Reactivity |
|---|---|---|---|---|---|
| 2:40 | | | The twins are eating the remaining tuwon dawa the left earlier | dawa with dried baobab leaves soup | |
| 2:45 | BB | | Baby still sleeping | | |
| 2:50 | BB | | Baby wakes up and sat next to the mother | | |
| 2:55 | SS | BB | The sister brought millet pap to the baby, the baby is drinking the pap | Cold millet pap | |
| 3:00 | BB | | Baby drinking the pap | Cold semi liquid pap | |
| 3:05 | BB,MM | | The baby is using her fingers to leak the cup and the mother is sleeping | | |
| 3:10 | BB | | The baby is playing while the mother is sleeping | | |
| 3:15 | | | Baby playing with the sand | | |
| 3:20 | FS | | The first son came back from the stream, he bath and washes his clothes there, fetches water in a bowl, dressed up and went out. | | |
| 3:25 | BB,YB | | The baby is playing with her brother | | |
| 3:30 | | | Baby playing | | |
| 3:35 | MM | | Mother still sleeping | | |
| 3:40 | BB,SS | | The baby and her sister went to the neighbor house | | |
| 3:45 | BB | | The baby is eating shea butter | Shea butter | |
| 3:50 | BB | | The baby is playing with the seed on the ground and sand.<br>The baby is drinking water | | |
| 3:55 | BB | | The baby is wearing my shoes | Wearing | |

| TIME | ACTOR INITIALS | RECIPIENT INITIALS | WHAT HAPPENED | FOOD | Reactivity |
|---|---|---|---|---|---|
| | | | | my shoes | |
| 4:00 | BB | JS | The baby is playing with her sister, running round the house | | |
| 4:05 | BB | | The baby is playing, jumping and shouting, the mother beats her but she was laughing | | |
| 4:10 | BB | | The baby is pissing up on her legs, the mother beats her and she started crying, she put water on her body, removed her pants and dry them | She looks at me and beat her | |
| 4:15 | BB | | The baby is eating the shea butter fruit she took from the ground | Shea butter fruit | |
| 4:20 | FS | | The first son is carving wood into the hoe | | |
| 4:25 | BG | | The big girl is washing plates, cups and bowls | | |
| 4:30 | BG | | The big girl carries clothes in a big container and went out. The mother said she is going to wash them in the stream | | |
| 4:35 | BB,JS | | The baby and her sister are playing with other children under the mango tree with sand, stone and shea butter seed | | |
| 4:40 | | | The baby is still playing | | |
| 4:45 | BB | | The bay is drinking water from the pot with a cup.<br>The baby started crying following the mother | | |
| 4:50 | MM | BB | Mother gave the baby pap in a bowl and she starts drinking | pap | |
| 4:55 | YB | | The small boy is preparing tuwon dawa, water and sugar mixing them together and he drink<br>The baby wants to drink and he gave her | Tuwon dawa mixed with water | |
| 5:00 | BB | | The baby is still drinking | | |

| TIME | ACTOR INITIALS | RECIPIENT INITIALS | WHAT HAPPENED | FOOD | Reactivity |
|---|---|---|---|---|---|
| | | | | | |
| 5:05 | BB | | The baby is playing with her doll baby | | |
| 5:10 | BB | | The baby is playing with her doll baby on her mother's lap | | |
| 5:15 | | | The baby is playing with the twins sitting on the sand | | |
| 5:20 | BB | | The baby is drinking water from the kettle | | |
| 5:25 | MM | | Mother clearing the fire place.<br>Mother enter the toilet | | |
| 5:30 | MM,BB | | Mother giving the baby shea butter fruit.<br>Mother setting the fire | Shea butter fruit | |
| 5:35 | MM | | Still setting the fire | | |
| 5:40 | MM | | Mother adds water into the pot and put on fire.<br>She is sieving flour | | |
| 5:45 | MM,BG | | Still sieving the flour, the big girl came back from the stream, she spread the clothes and rub Vaseline on her body | | |
| 5:50 | BG,SS<br>BB | | The big girl is sweeping the room and the surrounding.<br>The sister helps her sister in sweeping the compound.<br>The baby is playing | | |
| 5:55 | FF,MM | | The father comes back home on a bicycle with the bunch of wood at the back.<br>Mother took a bucket of water to the toilet with sponge and soap and say to the father the water is ready.<br>Father enters the toilet | | |
| 6:00 | MM,FF | BB | Mother gives pap to the baby in a bowl.<br>The father is reciting the Qur'an | | |

Primary Questions (Complete after you complete data collection):

From what you have seen observing THIS HOUSEHOLD, please answer the following questions in detail:

| Question | Write your detailed response here |
|---|---|
| 1. In this household, what would be the best way(s) to give nutritional supplements such as an MNP to young children to ensure compliance?<br><br>Why? | Through the mother and the elder sister because they are the one's taking care of the children, the sister help the mother in taking care of the little ones and other household chores |
| 2. In this household, who is favored during meal preparation or feeding?<br><br>How is this favoritism expressed? | I do not notice any favoritism during feeding in this household because whoever wants to eat takes what will be enough for him or her.<br>The only favoritism is based on age because what the older ones can eat the younger ones cannot eat |
| 3. How could we promote the MNP supplement for this family that would attract their attention?<br><br>Who would they listen to? | Through the mother and the father, I mentioned the mother first because since when I enter this house, I didn't meet the father until when am about to leave the house that he pop in. the mother explain to him, that is all; from this I can say that the mother can make decision on the family when the father is not around without any problem. Also when we are coming into the community, we do not come through the village head, therefore, each family have their own decision on their family |
| 4. In this household, what would be the best ways to promote the MNP but limit how much it would be shared?<br><br>Why? | Through proper sensitization of the mother about the MNP since the mother is in charge of meal preparation and feeding |
| 5. What other issues and concerns did you see with this family that are important for us to consider with regard to introduction of an MNP?<br><br>Why? | Lack of hygiene, all the children in the house eat without washing their hands including the elder ones even when the finished playing with sand.<br>Everybody in the house walk bear footed including the mother.<br>Inadequate food because throughout my staying in the house it is only tuwon dawa that was eaten and prepared and kunu (millet pap) |

| | |
|---|---|
| 6. Based on your experience observing this child today, do you believe reactivity negatively impacted your ability to see the food-related behaviors of the child?<br><br>Please explain your answer in detail. | I don't think reactivity impacted on my ability to see food related behavior of the child because all the reactivity that happened are not food related |
